# Beta-blocker use and cardiovascular event risk in patients with heart failure with preserved ejection fraction

**DOI:** 10.1038/s41598-018-27799-y

**Published:** 2018-06-22

**Authors:** Tetsuro Tsujimoto, Hiroshi Kajio

**Affiliations:** Department of Diabetes, Endocrinology, and Metabolism, Center Hospital, National Center for Global Health and Medicine, Tokyo, Japan

## Abstract

To assess whether beta-blocker use is associated with cardiovascular events and mortality in patients with heart failure with preserved ejection fraction (HFpEF), this study analyzed the Treatment of Preserved Cardiac Function Heart Failure with an Aldosterone Antagonist (TOPCAT) trial data using Cox proportional hazard models. Adjusted HRs for composite cardiovascular events in all patients and in patients without previous MI were significantly higher for those on beta-blockers than for those not on beta-blockers (Hazard ratio [HR] for all patients 1.23, 95% confidence interval [95% CI] 1.02–1.49; HR for patients without previous MI 1.35, 95% CI 1.08–1.70), whereas that for patients with previous MI was not significantly different (HR 1.06, 95% CI 0.74–1.54). Additionally, cardiovascular event risk in propensity score-matched patients without previous MI was significantly higher in those on beta-blockers than in those not on beta-blockers. Risks of all-cause death, major cardiovascular events, and heart failure hospitalization were significantly higher in those on beta-blockers than in those not on beta-blockers. Beta-blocker use in HFpEF patients, particularly those without previous MI, was associated with increased risk of unfavorable cardiovascular events.

## Introduction

Heart failure with preserved ejection fraction (HFpEF) accounts for approximately half of heart failure cases^1–3^. In epidemiological studies, rates of all-cause mortality and hospitalization are similar between patients with HFpEF and heart failure with reduced ejection fraction (HFrEF)^1,3^. The prevalence of HFpEF has increased over time, but mortality has remained unchanged^1^. The benefit of medical therapies for heart failure has been limited to heart failure with reduced ejection fraction (HFrEF); favorable evidence from clinical trials of HFpEF is lacking. Therefore, no clinical guideline has offered specific recommendations for the management of HFpEF^4,5^.

Beta-blockers reduce the risk of hospitalization and mortality in HFrEF patients^5,6^. Beta-blockers also significantly reduce recurrent myocardial infarction (MI) and mortality in patients with previous MI^7,8^. However, there is little evidence for the efficacy of beta-blockers in HFpEF patients^9^. In addition, there is no study reporting the efficacy of beta-blockers in HFpEF patients who do not have a history of MI. The present study aimed to assess whether beta-blocker use is associated with cardiovascular events and mortality in HFpEF patients. We also assessed whether beta-blockers are effective in HFpEF patients with and without previous MI.

## Methods

### Study patients and design

We used the Treatment of Preserved Cardiac Function Heart Failure with an Aldosterone Antagonist (TOPCAT) trial data to assess the association between beta-blocker use and cardiovascular events and mortality in HFpEF patients^10^. A detailed information about the study design, protocol, and patient characteristics has been reported previously^11,12^. In brief, TOPCAT was an international, multicenter, randomized trial. A total of 3,445 patients in 6 countries were randomly assigned to receive spironolactone (n = 1,722) or a placebo (n = 1,723) from August 10, 2006, to January 31, 2012. Patients 50 years or older were enrolled if they had at least one symptom and one sign of heart failure on a prespecified list of symptoms (dyspnea on mild or moderate exertion, orthopnea, or paroxysmal nocturnal dyspnea) and signs (jugular venous pressure ≥10 cm H_2_O, any rales post cough, lower edema, or chest x-ray demonstrating cardiomegaly, pulmonary congestion, or pleural effusion), a left ventricular ejection fraction (LVEF) ≥45%, controlled systolic blood pressure, and a serum potassium level <5.0 mmol/L. Eligibility criteria included a history of hospitalization for heart failure in the past 12 months or an elevated level of natriuretic peptide in the last 60 days before randomization. Exclusion criteria included severe pulmonary disease, severe systemic illness,; heart transplant, pericardial constriction or obstructive cardiomyopathy,, severe renal dysfunction; or chronic hepatic disease^12^. The mean follow-up interval was 3.3 years in both groups. This study was approved by the institutional review board of the National Center for Global Health and Medicine. The National Heart, Lung, and Blood Institute (NHLBI) approved our data use. This study was conducted in accordance with the Declaration of Helsinki. All methods were conducted in accordance with the relevant guidelines and regulations. Because the present study was a post-hoc analysis of the TOPCAT trial, the present study received the ethical approval for the use of an opt-out method of obtaining informed consent. The patients were anonymized by NHLBI before our data use.

### Outcome measurements

The primary outcome was a composite event of cardiovascular death, aborted cardiac arrest, nonfatal stroke, nonfatal MI, or heart failure hospitalization. The secondary outcome was all-cause death, major cardiovascular events, or heart failure hospitalization. Major cardiovascular events included cardiovascular death, nonfatal stroke, and nonfatal MI. Cardiovascular and non-cardiovascular mortality was assessed to analyze mortality in more detail. According to prespecified criteria, a clinical end-point committee at Brigham and Women’s Hospital adjudicated all events^11^. Patients were assessed every four months during first year in the study and every six months thereafter^10^.

### Confounders

Potential confounders were extracted at baseline: sex, age, race and ethnicity (white, black, asian, or others), alcohol intake (0, 1–5, 6–10, or ≥11 drinks per week), smoking status (current smoker, former smoker, or never smoked), body mass index, New York Heart Association (NYHA) functional classification, history of hypertension, diabetes, dyslipidemia, cardiovascular events (MI, angina pectoris, stroke, heart failure hospitalization, peripheral arterial disease, atrial fibrillation, coronary artery bypass graft surgery, percutaneous coronary intervention, pacemaker, or implanted cardioverter defibrillator), chronic obstructive pulmonary disease, or asthma, medications (angiotensin II receptor blockers or angiotensin-converting enzyme inhibitors, diuretics, calcium channel blockers, statins, or aspirin), randomization arm (placebo or spironolactone), systolic and diastolic blood pressure, estimated glomerular filtration rate, heart rate, and region of enrollment (United States, Canada, Brazil, Argentina, Russia, and Georgia).

### Statistical analysis

Demographic data are shown as means ± standard deviations (SDs) or proportions (%). Patients were classified into two groups: those on and not on beta-blockers. Student’s *t*-tests were used to compare continuous variables, and chi-squared tests were used to compare categorical variables. Kaplan–Meier survival curves were constructed for outcomes in patients on and not on beta-blockers. Cox proportional hazard analyses were performed to calculate hazard ratios (HRs) and 95% confidence intervals (CIs) for outcomes in patients on beta-blockers compared with those of patients not on beta-blockers. We included all potential confounders listed above for adjustment. Further adjustment as a sensitivity analysis was made to add health state to those confounders. Health state was evaluated using a visual analog scale (the best status corresponds to 100 and the worst status corresponds to 0). The outcomes were further analyzed in patients with and without previous MI. Because beta-blocker use in patients with diabetes is controversial^13–17^, we conducted further analyses for outcomes in patients with and without diabetes.

For another sensitivity analysis, Cox proportional hazard analysis was performed to assess outcomes in propensity score-matched patients without previous MI who were on or not on beta-blockers^18^. To match baseline characteristics, 1:1 nearest neighbor matching were used without replacement. Because the number of patients with previous MI was small, we did not analyze HRs of outcomes in those patients. The propensity score was used adjusting for confounders and estimated the probability that patients would have been assigned to beta-blocker use^19,20^. The propensity score was derived using a logistic regression model including potential confounders and health state as predictors and beta-blocker use as the outcome variable. Using overall TOPCAT data, we performed an additional analysis with adjustment for propensity scores as a covariate^21^. Standardized differences <0.10 were not considered meaningfulwell-balanced^18^.

Statistical analyses were performed with Stata software (version 14.1, Stata Corp., College Station, Texas, USA). *P* values < 0.05 were considered statistically significant.

## Results

### Baseline characteristics

The present study included 3,417 HFpEF patients, 887 with and 2,530 without previous MI. Baseline characteristics are presented in Table 1. Compared with patients not on beta-blockers, those on beta-blockers were associated with a younger age; smaller female proportion; higher prevalence of hypertension, dyslipidemia, previous MI, and angina pectoris; more patients took coronary artery bypass graft surgery or previous treatment with percutaneous coronary intervention; more use of aspirin and statins; lower prevalence of chronic obstructive pulmonary disease and asthma; and less use of calcium channel blockers. The distribution of patients according to NYHA functional classification was not significantly different between those on and not on beta-blockers. Similar characteristics were found in patients with and without previous MI.

**Table 1:** Baseline characteristics of patients with heart failure with preserved ejection fraction on and not on beta-blockers*.

	All			MI (−)			MI ( + )		
	β (−)	β (+)	P value	β (−)	β (+)	P value	β (−)	β (+)	P value
	n = 760	n = 2,657		n = 602	n = 1,928		n = 158	n = 729	
Age (years)	69.5 (9.8)	68.3 (9.5)	0.002	69.6 (9.9)	68.6 (9.6)	0.02	69.1 (9.2)	67.5 (9.3)	0.05
Female sex (%)	54.9	50.7	0.04	58.5	56.2	0.24	41.1	36.1	0.23
**Race and ethnicity (%)**									
White	90.0	88.5	0.23	88.0	86.8	0.43	97.5	92.7	0.02
Black	7.6	8.9	0.26	9.2	10.4	0.36	1.9	4.9	0.09
Asian	0.9	0.4	0.12	1.0	0.4	0.06	0.6	0.7	0.94
Others	1.5	2.2	0.20	1.8	2.4	0.42	0.0	1.7	0.10
**Smoking status (%)**									
Never	53.9	52.4	0.46	56.5	56.6	0.96	44.3	41.4	0.50
Former	35.3	37.3	0.31	34.0	35.0	0.66	39.9	43.2	0.44
Current	10.8	10.3	0.70	9.5	8.4	0.41	15.8	15.4	0.88
**Alcohol drinks/week (%)**									
0	76.7	78.3	0.34	77.9	77.7	0.91	72.2	80.0	0.03
1–5	17.3	16.8	0.77	16.5	17.5	0.53	20.2	14.8	0.08
6–10	5.1	3.2	0.01	4.8	3.1	0.04	6.3	3.7	0.13
11–	0.9	1.7	0.14	0.8	1.7	0.12	1.3	1.5	
**NYHA functional classification (%)**									
I/II	68.6	66.6	0.30	69.6	67.7	0.37	64.6	63.7	0.82
III/IV	31.4	33.4		30.4	32.3		35.4	36.4	
Body mass index (kg/m^2^)^†^	31.8 (7.4)	32.1 (7.0)	0.34	32.1 (7.7)	32.3 (7.2)	0.56	30.7 (5.9)	31.5 (6.3)	0.14
Diabetes (%)	29.7	33.2	0.07	29.4	31.5	0.33	31.0	37.6	0.12
Hypertension (%)	88.6	92.3	0.001	88.7	91.7	0.02	88.0	94.0	0.008
Dyslipidemia (%)	53.4	62.1	<0.001	49.0	56.2	0.002	70.3	77.9	0.03
**History of cardiovascular events (%)**									
Myocardial infarction	20.8	27.4	<0.001	—	—		—	—	
Angina pectoris	48.3	42.8	0.008	34.1	39.0	0.03	76.0	72.8	0.42
Stroke	8.4	7.5	0.41	7.0	6.1	0.42	13.9	11.4	0.37
Peripheral arterial disease	9.2	9.2	0.99	7.1	7.1	0.97	17.1	14.8	0.47
Hospitalization for heart failure	71.2	72.8	0.39	69.8	73.2	0.09	76.6	71.5	0.19
Atrial fibrillation	37.5	34.6	0.14	37.5	37.8	0.90	37.3	26.2	0.005
Percutaneous coronary intervention	10.4	15.8	<0.001	5.7	8.0	0.05	28.5	36.2	0.06
CABG surgery	7.5	14.3	<0.001	4.5	8.7	0.001	19.0	29.1	0.01
Implanted cardioverter defibrillator	0.7	1.5	0.08	0.5	1.1	0.19	1.3	2.5	0.35
Pacemaker	8.3	7.6	0.55	8.1	7.5	0.61	8.9	8.0	0.70
COPD (%)	15.7	10.4	<0.001	15.0	10.0	0.001	18.4	11.7	0.02
Asthma (%)	9.3	5.6	<0.001	9.6	5.7	0.001	8.2	5.5	0.18
**Medications (%)**									
ACE-I/ARB	84.7	84.1	0.66	84.9	83.5	0.40	84.2	85.7	0.61
Calcium channel blockers	50.8	33.8	<0.001	51.2	34.2	<0.001	49.4	32.8	<0.001
Diuretics	79.3	82.5	0.05	81.9	85.5	0.03	69.6	74.5	0.20
Aspirin	58.2	67.6	<0.001	53.5	62.0	<0.001	76.0	82.3	0.06
Statin	38.4	56.3	<0.001	34.1	49.6	<0.001	55.1	73.9	<0.001
**Randomization arm**									
Spironolactone (%)	49.0	50.4	0.49	49.0	50.5	0.51	48.7	49.9	0.78
Estimated GFR(mL/min/1.73 m^2^)	68.5 (19.9)	67.4 (20.1)	0.19	69.9 (20.4)	67.2 (19.9)	0.08	67.0 (17.9)	67.9 (20.7)	0.61
Systolic blood pressure (mmHg)	129.5 (13.2)	129.1 (14.1)	0.49	129.9 (13.5)	129.9 (14.1)	0.89	128.0 (11.9)	127.3 (13.8)	0.54
Diastolic blood pressure (mmHg)	76.5 (10.4)	75.6 (10.7)	0.05	76.7 (10.4)	76.0 (10.9)	0.18	75.8 (10.3)	74.6 (10.1)	0.20
Heart rate (beats per minute)	69.4 (10.6)	69.0 (10.3)	0.28	70.0 (10.5)	69.7 (10.6)	0.55	67.3 (10.9)	67.0 (9.4)	0.76
**Region of enrollment (%)**									
United States	27.0	34.9	<0.001	29.4	37.5	<0.001	17.7	28.0	0.008
Canada	9.1	9.6	0.67	8.7	9.6	0.48	10.8	9.6	0.65
Russia	33.4	30.5	0.12	27.7	23.7	0.04	55.1	48.4	0.13
Republic of Georgia	17.6	18.0	0.82	19.1	20.9	0.33	12.0	10.3	0.52
Brazil	8.3	3.7	<0.001	9.3	4.4	<0.001	4.4	1.9	0.06
Argentina	4.6	3.3	0.09	5.8	3.9	0.04	0.0	1.8	0.09

^*^Data are presented as number of participants, percent, or mean (standard deviation).

^†^Body mass index was calculated as weight in kilograms divided by the square of height in meters. NYHA, New York heart association; CABG, coronary artery bypass graft; COPD, chronic obstructive pulmonary disease; ACE-I, angiotensin-converting enzyme inhibitors; ARB, angiotensin II receptor blockers; GFR, glomerular filtration rate; MI, myocardial infarction; β, beta blockers.

### Beta-blocker use and risk of cardiovascular events

**Figure 1:**
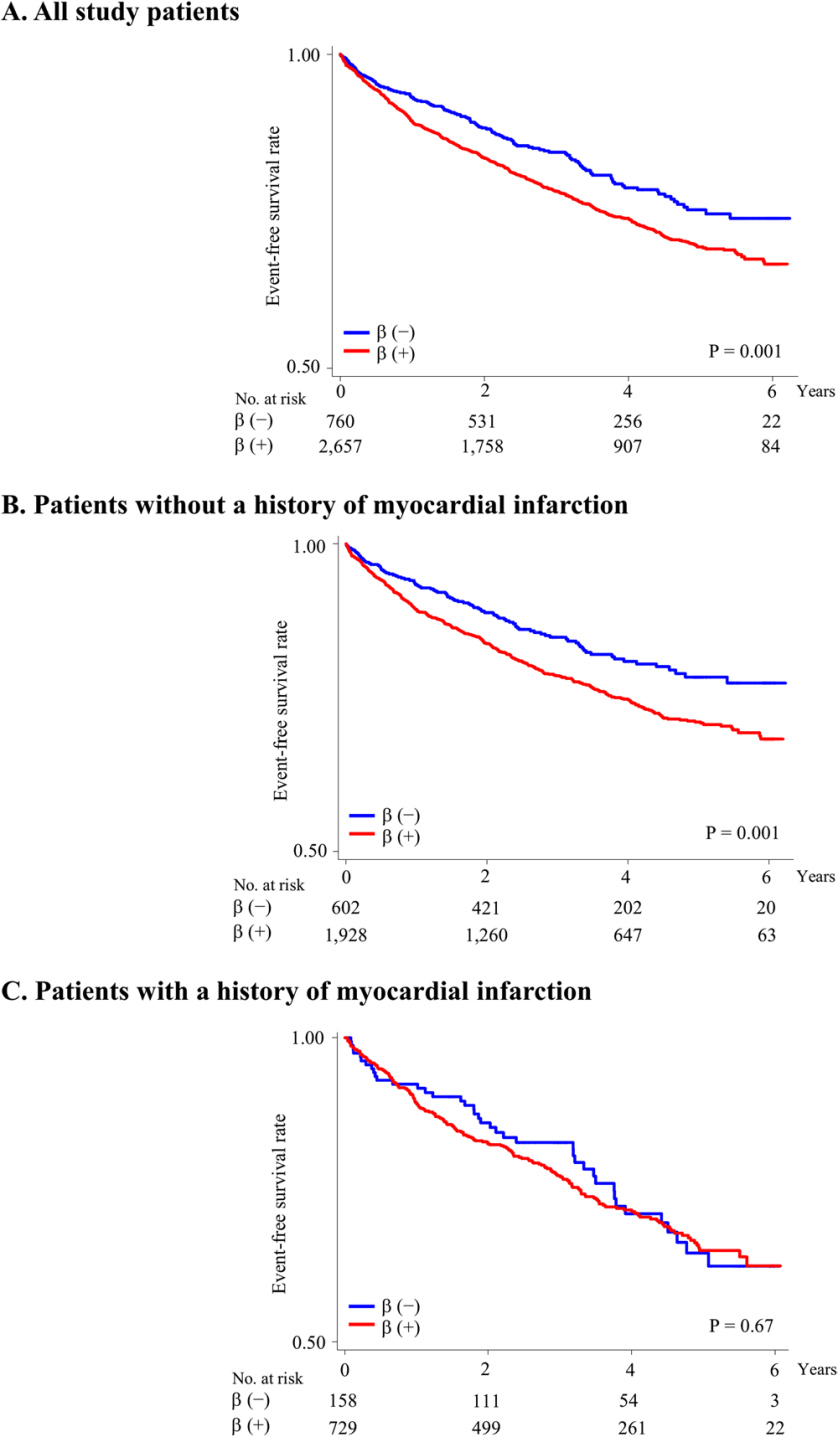
Kaplan–Meier survival curves for primary outcome events. Rates of freedom from primary outcome events in all study patients (**A**), patients without a history of MI (**B**), and patients with a history of MI (**C**). The primary outcome was a composite of cardiovascular death, aborted cardiac arrest, nonfatal MI, nonfatal stroke, or hospitalization for the management of heart failure. β, beta-blockers; MI, myocardial infarction.

The mean (SD) follow-up period was 3.3 (1.7) years; 778 patients had primary outcome event. Cumulative event rates and Kaplan–Meier survival curves for primary outcome events are presented in Table 2 and Fig. 1, respectively. Primary outcome event rates (number per 1,000 person-years) in patients on and not on beta-blockers were 78.5 and 58.1, respectively. The primary outcome event risk was significantly higher in those on beta-blockers than in those not on beta-blockers (unadjusted HR 1.35, 95% CI 1.12–1.62, *P* = 0.001) (Fig. 1A). The primary outcome event risk in patients without previous MI was significantly higher in those on beta-blockers than in those not on beta-blockers (unadjusted HR 1.44, 95% CI 1.16–1.80, *P* = 0.001) (Fig. 1B), whereas that in patients with previous MI was not significantly different between those on and not on beta-blockers (unadjusted HR 1.07, 95% CI 0.77–1.51, *P* = 0.67) (Fig. 1C). Adjusted HR for primary outcome events in all study patients and in patients without previous MI was also significantly higher for those on beta-blockers than for those not on beta-blockers (adjusted HR in all patients 1.26, 95% CI 1.04–1.53, *P* = 0.02 and adjusted HR in patients without previous MI 1.39, 95% CI 1.11–1.75, *P* = 0.005), whereas that for patients with previous MI did not differ significantly between those on and not on beta-blockers (adjusted HR 1.08, 95% CI 0.75–1.57, *P* = 0.67). Similar results were observed after further adjustments with health state (adjusted HR in all patients 1.26, 95% CI 1.04–1.54, *P* = 0.01; adjusted HR in patients without previous MI 1.40, 95% CI 1.11–1.76, *P* = 0.004; and adjusted HR in patients with previous MI 1.08, 95% CI 0.74–1.57, *P* = 0.67). The analysis limited to patients without previous MI but had angina pectoris showed a higher risk of primary outcome events in those on beta-blockers (adjusted HR 1.67, 95% CI 1.02–2.73, *P* = 0.04). There was a significant interaction between beta-blocker use and MI history in the multivariable model (*P* value for interaction term <0.001).

**Table 2:** Primary and secondary outcome events in patients on and not on beta-blockers*.

	All			MI (−)			MI (+)		
	β (−)	β (+)	P value	β (−)	β (+)	P value	β (−)	β (+)	P value
	n = 760	n = 2,657		n = 602	n = 1,928		n = 158	n = 729	
**Event**									
**Primary outcome events** ^†^									
No. of patients	138	640		98	444		40	196	
Event rate (per 1,000 person-year)	58.1	78.5		52.3	75.5		79.6	86.2	
Unadjusted HR (95% CI)	1.00 (ref)	1.35 (1.12–1.62)	0.001	1.00 (ref)	1.44 (1.16–1.80)	0.001	1.00 (ref)	1.07 (0.77–1.51)	0.67
Adjusted HR (95% CI)	1.00 (ref)	1.26 (1.04–1.53)	0.02	1.00 (ref)	1.39 (1.11–1.75)	0.005	1.00 (ref)	1.08 (0.75–1.57)	0.67
**All-cause death**									
No. of patients	102	418		73	286		29	132	
Event rate (per 1,000 person-year)	40.2	46.4		36.7	44.2		52.8	52.1	
Unadjusted HR (95% CI)	1.00 (ref)	1.15 (0.93–1.43)	0.19	1.00 (ref)	1.16 (0.90–1.49)	0.15	1.00 (ref)	0.99 (0.66–1.48)	0.95
Adjusted HR (95% CI)	1.00 (ref)	1.19 (0.95–1.50)	0.12	1.00 (ref)	1.28 (0.97–1.67)	0.07	1.00 (ref)	1.03 (0.66–1.61)	0.89
**Cardiovascular death**									
No. of patients	66	267		44	179		7	44	
Event rate (per 1,000 person-year)	26.0	29.6		22.1	27.6		12.7	17.3	
Unadjusted HR (95% CI)	1.00 (ref)	1.14 (0.87–1.49)	0.34	1.00 (ref)	1.25 (0.90–1.73)	0.18	1.00 (ref)	0.87 (0.54–1.38)	0.54
Adjusted HR (95% CI)	1.00 (ref)	1.18 (0.89–1.57)	0.25	1.00 (ref)	1.29 (0.92–1.83)	0.14	1.00 (ref)	1.04 (0.62–1.77)	0.87
**Non-cardiovascular death**									
No. of patients	36	151		29	107		87	211	
Event rate (per 1,000 person-year)	14.2	16.8		14.6	16.5		44.0	51.2	
Unadjusted HR (95% CI)	1.00 (ref)	1.18 (0.82–1.70)	0.37	1.00 (ref)	1.13 (0.75–1.71)	0.54	1.00 (ref)	1.37 (0.62–3.04)	0.43
Adjusted HR (95% CI)	1.00 (ref)	1.25 (0.85–1.83)	0.26	1.00 (ref)	1.26 (0.82–1.96)	0.28	1.00 (ref)	0.99 (0.40–2.41)	0.98
**Major cardiovascular events** ^‡^									
No. of patients	90	411		62	268		28	143	
Event rate (per 1,000 person-year)	36.2	47.1		31.9	42.4		51.7	59.3	
Unadjusted HR (95% CI)	1.00 (ref)	1.30 (1.03–1.63)	0.02	1.00 (ref)	1.33 (1.01–1.75)	0.04	1.00 (ref)	1.15 (0.77–1.72)	0.50
Adjusted HR (95% CI)	1.00 (ref)	1.30 (1.02–1.65)	0.03	1.00 (ref)	1.35 (1.01–1.81)	0.04	1.00 (ref)	1.34 (0.86–2.09)	0.20
**Hospitalization for heart failure**									
No. of patients	69	375		48	277		21	98	
Event rate (per 1,000 person-year)	28.6	45.0		25.1	46.3		41.4	41.5	
Unadjusted HR (95% CI)	1.00 (ref)	1.58 (1.22–2.04)	<0.001	1.00 (ref)	1.85 (1.36–2.51)	<0.001	1.00 (ref)	1.00 (0.62–1.60)	0.99
Adjusted HR (95% CI)	1.00 (ref)	1.37 (1.05–1.79)	0.02	1.00 (ref)	1.67 (1.22–2.30)	0.001	1.00 (ref)	0.84 (0.49–1.43)	0.52

^*^Data are presented as number or hazard ratio (95% CI).

^†^The primary outcome was a composite of cardiovascular death, aborted cardiac arrest, nonfatal myocardial infarction, nonfatal stroke, or hospitalization for the management of heart failure.

^‡^Major cardiovascular events included all-cause death, nonfatal myocardial infarction, and nonfatal stroke.

HFpEF, heart failure with preserved left ventricular ejection fraction; MI, myocardial infarction; β, beta blockers; CI, confidence interval; HR, hazard ratio.

All-cause mortality in all patients, patients without previous MI, and patients with previous MI was not significantly higher in those on beta-blockers than in those not on beta-blockers (adjusted HR in all patients 1.19, 95% CI 0.95–1.50, *P* = 0.12; adjusted HR in patients without previous MI 1.28, 95% CI 0.97–1.67, *P* = 0.07; adjusted HR in patients with previous MI 1.03, 95% CI 0.66–1.61, *P* = 0.89). However, rates of major cardiovascular events and hospitalization for heart failure in patients without previous MI were significantly higher in those on beta-blockers than in those not on beta-blockers (adjusted HR for major cardiovascular events 1.35, 95% CI 1.01–1.81, *P* = 0.04 and adjusted HR for heart failure 1.67, 95% CI 1.22–2.30, *P* = 0.001, respectively). Rates of major cardiovascular events and hospitalization for heart failure in patients with previous MI did not significantly differ between those on and not on beta-blockers.

Primary and secondary outcomes in patients with and without diabetes are presented in Supplemental Table 1. Risks of primary outcome events, major cardiovascular events, and hospitalization for heart failure in patients without diabetes were significantly higher in those on beta-blockers than in those not on beta-blockers (adjusted HR for primary outcome event 1.46, 95% CI 1.12–1.90, *P* = 0.005; adjusted HR for major cardiovascular events 1.39, 95% CI 1.01–1.80, *P* = 0.04; adjusted HR for heart failure 1.74, 95% CI 1.17–2.61, *P* = 0.007). Although the samples were limited, risks of primary and secondary outcome events in patients with diabetes did not differ significantly between those on and not on beta-blockers.

### Sensitivity analysis

**Figure 2:**
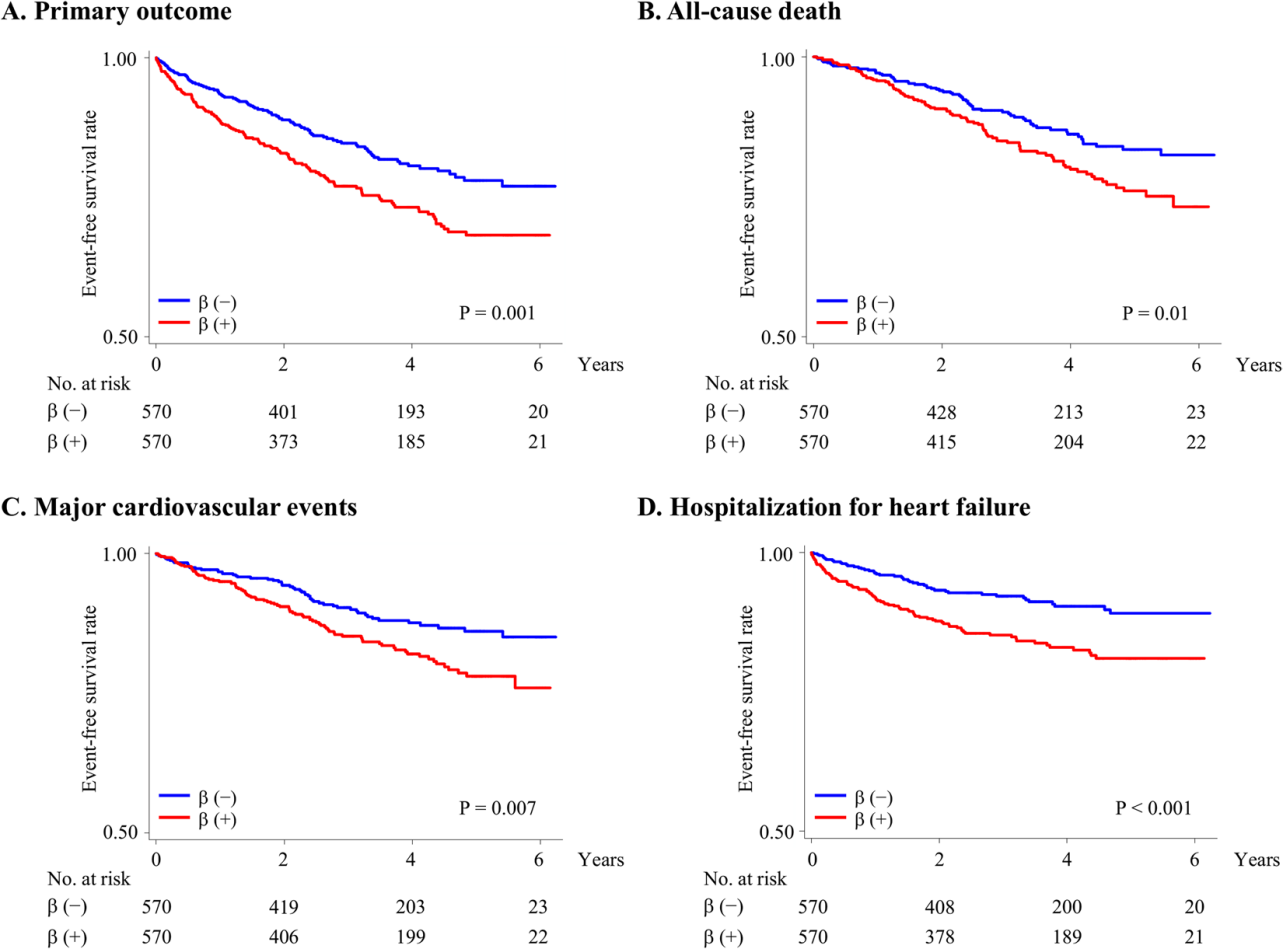
Primary and secondary outcomes in propensity score-matched patients without a history of MI. Rates of freedom from primary outcome events (**A**), all-cause death (**B**), major cardiovascular events (**C**), and hospitalization for heart failure (**D**). The primary outcome was a composite of cardiovascular death, aborted cardiac arrest, nonfatal MI, nonfatal stroke, or hospitalization for the management of heart failure. Major cardiovascular events included all-cause death, nonfatal MI, and nonfatal stroke. β, beta-blockers; MI, myocardial infarction.

**Figure 3:**
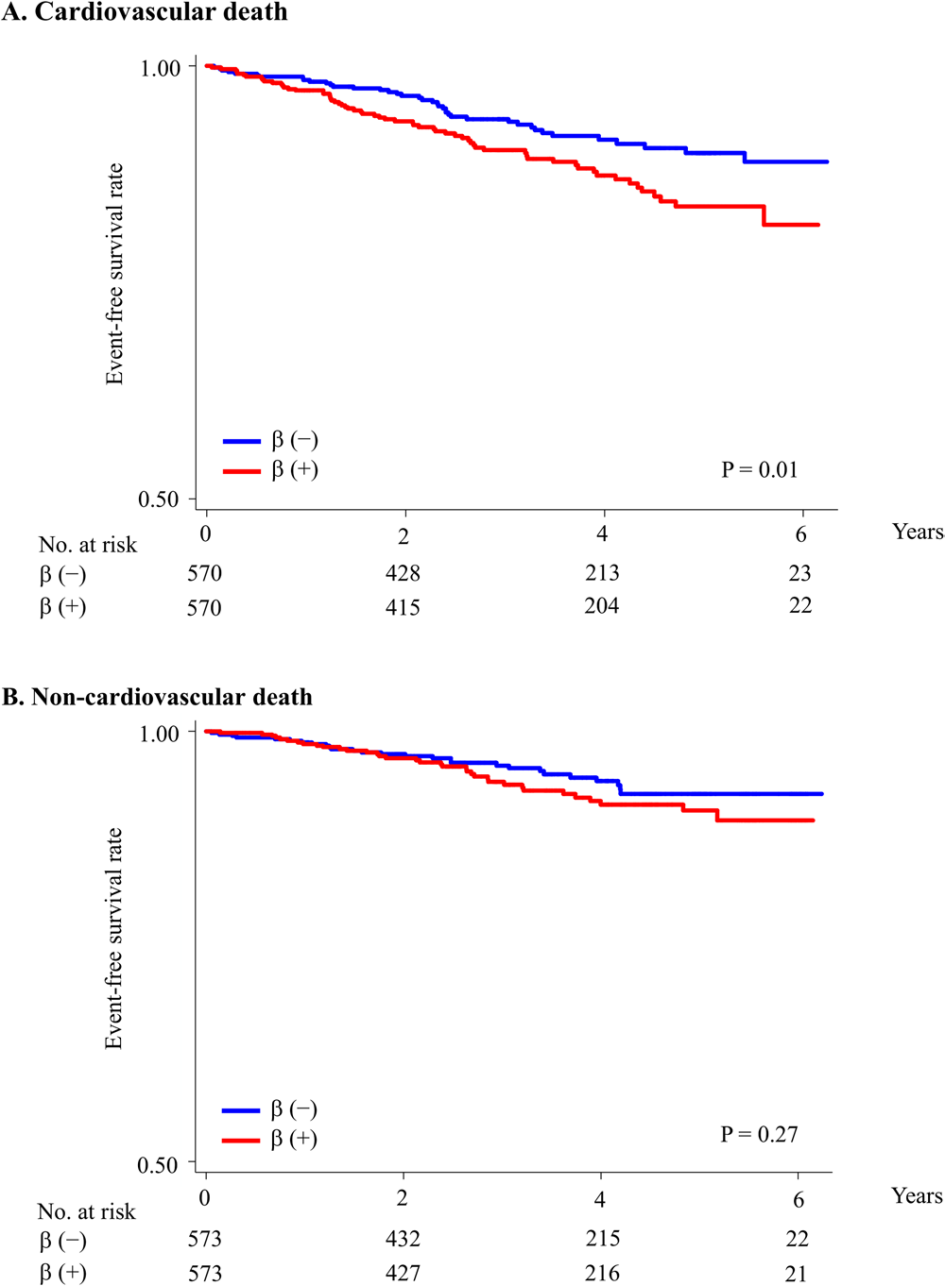
Cardiovascular and non-cardiovascular death in propensity score-matched patients without a history of MI. Rates of freedom from cardiovascular death (**A**) and non-cardiovascular death (**B**). β, beta-blockers; MI, myocardial infarction.

The baseline characteristics of 1,154 propensity score-matched patients without previous MI are shown in Supplemental Table 2. No significant difference was observed between those on and not on beta-blockers. The risk of primary composite events in propensity score-matched patients without previous MI was significantly higher in those on beta-blockers than in those not on beta-blockers (HR 1.55, 95% CI 1.19–2.01, *P* = 0.001) (Fig. 2A). The analysis with adjustment for propensity score as a covariate showed that the risk of primary outcome events in patients without previous MI was significantly higher in those on beta-blockers than in those not on beta-blockers (HR 1.37, 95% CI 1.09–1.72, *P* = 0.008). Risks of all-cause death, major cardiovascular events, and heart failure hospitalization were significantly higher in those on beta-blockers than in those not on beta-blockers (HR for all-cause death 1.50, 95% CI 1.10–2.04, *P* = 0.01; HR for cardiovascular events 1.58, 95% CI 1.14–2.20, *P* = 0.007; and HR for hospitalization for heart failure 1.95, 95% CI 1.37–2.79, *P* < 0.001) (Fig. 2B–D). Although non-cardiovascular mortality in patients without previous MI did not differ significantly between those on and not on beta-blockers, cardiovascular mortality was significantly higher in those on beta-blockers than in those not on beta-blockers (HR 1.61, 95% CI 1.08–2.40, *P* = 0.01) (Fig. 3).

## Discussion

In the present study, various analyses of HFpEF patients revealed that beta-blocker use was significantly associated with an increased risk of composite cardiovascular events. Particularly, in HFpEF patients without previous MI, beta-blocker use was significantly associated with a higher risk of composite cardiovascular events, whereas beta-blocker use in those with previous MI was not significantly associated with those events. Propensity score-matched analyses in HFpEF patients without previous MI found that beta-blocker use was associated with increased risks of all-cause death, major cardiovascular events, and heart failure hospitalization.

Sympathetic nervous system activation, ventricular diastolic dysfunction, and left ventricular hypertrophy are often observed in HFpEF patients^22,23^. Previous studies have suggested that beta-blocker use may not only reduce the effects of hypercatecholaminemia but also improve ventricular diastolic dysfunction and left ventricular hypertrophy^24,25^. Based on these findings, beta-blocker use may have cardioprotective effects in HFpEF patients. However, the efficacy of beta-blockers in HFpEF patients remains unclear. In addition, although beta-blocker use was reportedly associated with decreased risks of recurrent MI and death in patients with previous MI^7,8^, there is no study regarding the effect of beta-blockers in HFpEF patients without previous MI. In the present study, beta-blocker use was associated with an increased risk of composite cardiovascular events in HFpEF patients, particularly in those without previous MI, whereas such association was not observed in HFpEF patients with previous MI. A possible explanation is that disadvantages of beta-blockers, including weight gain and metabolic adverse effects, may outweigh their cardioprotective effects in HFpEF patients, particularly in those without previous MI^26,27^. Limited data available from previous studies on the effects of beta-blockers in HFpEF patients are conflicting^21,28–31^. Contrary to the results of our study, some studies reported beneficial effects of beta-blockers in HFpEF patients. In the subanalysis of the SENIORS trial, the effect of beta-blockers in patients with heart failure was similar in those with preserved and impaired ejection fraction^31^. In addition, a previous prospective study suggested that beta-blocker use was associated with lower all-cause mortality, but not with lower combined all-cause mortality or heart failure hospitalization^21^. A possible reason for these conflicting results was the definition of preserved ejection fraction. The former study^31^ included patients with an ejection fraction >35% (median ejection fraction: 46%), and >40% of the patients in the latter study^21^ had an ejection fraction of 40–49%; these values are extremely different from those in the TOPCAT study patients, who had an ejection fraction ≥45% (median ejection fraction: 56%). Beneficial effects of beta-blockers in these previous studies might be dependent on cardioprotective effects for ventricular dysfunction. Because of the lack of positive evidence, current guidelines do not recommend beta-blocker use for HFpEF patients^4,5^. For patients with preserved ejection fraction, particularly those without previous MI, further studies are warranted to reveal the effects of beta-blockers in HFpEF patients. In addition, although the present study defined HFpEF as left ventricular ejection fraction (LVEF) ≥45%, the new European guidelines make a different classification: the diagnosis of HFpEF requires LVEF ≥50%, and patients with LVEF 40–49% are considered to have heart failure with mid-range ejection fraction (HFmrEF)^4^. The pathophysiology is complicated, and HFpEF and HFmrEF are associated with different phenotypes including various cardiovascular and non-cardiovascular diseases^4^. Furthermore, no treatment has been shown to improve outcomes in each patient with HFpEF or HFmrEF. Because we could not fully evaluate the effects of beta-blockers in patients with LVEF ≥50% and those with LVEF 40%–49%, future studies are required to address these challenging issues.

The present study has several limitations to note. First, this study was a post-hoc analysis using data from the TOPCAT trial. The patients on beta-blockers were at high baseline risk, with more risk factors such as male sex, hypertension, and dyslipidemia. Even in patients without previous MI, those on beta-blockers had an increased prevalence of angina pectoris. Although we performed various analyses to adjust for potential confounders, unmeasured and unknown confounders may remain present, and statistical adjustment may not completely eliminate these confounders. We performed propensity score-matched analyses to minimize the effects of the potential confounders. However, it is also imperfect, and residual confounders may remain present. Furthermore, propensity-score matching may introduce another possibility of additional bias. To evaluate whether beta-blocker use is beneficial and safe in HFpEF patients, further randomized controlled trials are necessary. Second, because the number of HFpEF patients with previous MI was small, we could not perform sensitivity analysis of their results. Third, there was no rigorous definition of MI history and no detailed information about it; therefore, whether the severity of previous MI influences the results remains unknown. Forth, we could not clarify the types of beta-blockers. Thus, further large-scale studies are also needed to reveal the effects of different types of beta-blockers in HFpEF patients.

The present study demonstrated that beta-blocker use in HFpEF patients was associated with an increased risk of composite cardiovascular events. In particular, beta-blocker use in HFpEF patients without previous MI was associated with higher risks of all-cause death, major cardiovascular events, and heart failure hospitalization. To reveal indications for beta-blockers in HFpEF patients, further trials are required.

## Electronic supplementary material

Supplemental tables
