# Supplementary material for: Beta-blocker use and cardiovascular event risk in patients with heart failure with preserved ejection fraction
Source: Sci Rep. 2018 Jun 22;8:9556. doi: 10.1038/s41598-018-27799-y (PMC6015007; doi:10.1038/s41598-018-27799-y)
Supplement: Supplementary file 1 — Supplemental tables [file 41598_2018_27799_MOESM1_ESM.docx]

**Title:**

**Beta-blocker use and cardiovascular event risk in patients with heart failure with preserved ejection fraction**

Tetsuro Tsujimoto, M.D., Ph.D.^1^ Hiroshi Kajio, M.D., Ph.D.^1^

^1^Department of Diabetes, Endocrinology, and Metabolism, Center Hospital, National Center for Global Health and Medicine, Tokyo, Japan

**Address for correspondence:**

Tetsuro Tsujimoto, M.D., Ph.D.

Department of Diabetes, Endocrinology, and Metabolism,

Center Hospital, National Center for Global Health and Medicine, Tokyo, Japan

1-21-1 Toyama, Shinjuku-ku, Tokyo, 162-8655, Japan

Tel: +81-3-3202-7181

Fax: +81-3-3207-1038

E-mail: ttsujimoto@hosp.ncgm.go.jp

**Supplemental Table 1. Cardiovascular events and death in diabetic and non-diabetic patients with myocardial infarction on and not on beta-blockers***

|  |  |  | Diabetes |  |  |  | Non-diabetes |  |
| --- | --- | --- | --- | --- | --- | --- | --- | --- |
|  |  | β (−)  n = 226 | β (+)  n = 881 | P value |  | β (−)  n = 534 | β (+)  n = 1,776 | P value |
| **Event** |  |  |  |  |  |  |  |  |
| **Primary outcome events**^†^ |  |  |  |  |  |  |  |  |
| No. of patients |  | 64 | 305 |  |  | 74 | 335 |  |
| Event rate (per 1,000 person-year) |  | 105.5 | 130.4 |  |  | 41.9 | 57.6 |  |
| Unadjusted HR (95% CI) |  | 1.00 (ref) | 1.24 (0.95–1.62) | 0.11 |  | 1.00 (ref) | 1.37 (1.07–1.77) | 0.01 |
| Adjusted HR (95% CI) |  | 1.00 (ref) | 1.14 (0.86–1.52) | 0.36 |  | 1.00 (ref) | 1.46 (1.12–1.90) | 0.005 |
|  |  |  |  |  |  |  |  |  |
| **All-cause death** |  |  |  |  |  |  |  |  |
| No. of patients |  | 43 | 176 |  |  | 59 | 242 |  |
| Event rate (per 1,000 person-year) |  | 62.9 | 63.6 |  |  | 31.8 | 38.8 |  |
| Unadjusted HR (95% CI) |  | 1.00 (ref) | 1.01 (0.72–1.40) | 0.97 |  | 1.00 (ref) | 1.22 (0.92–1.62) | 0.17 |
| Adjusted HR (95% CI) |  | 1.00 (ref) | 1.13 (0.79–1.63) | 0.49 |  | 1.00 (ref) | 1.25 (0.93–1.71) | 0.13 |
| **Cardiovascular death** |  |  |  |  |  |  |  |  |
| No. of patients |  | 27 | 107 |  |  | 39 | 160 |  |
| Event rate (per 1,000 person-year) |  | 39.5 | 38.7 |  |  | 21.0 | 25.6 |  |
| Unadjusted HR (95% CI) |  | 1.00 (ref) | 0.98 (0.64–1.49) | 0.91 |  | 1.00 (ref) | 1.22 (0.86–1.73) | 0.27 |
| Adjusted HR (95% CI) |  | 1.00 (ref) | 1.16 (0.74–1.83) | 0.51 |  | 1.00 (ref) | 1.30 (0.89–1.89) | 0.17 |
| **Non-cardiovascular death** |  |  |  |  |  |  |  |  |
| No. of patients |  | 16 | 69 |  |  | 20 | 82 |  |
| Event rate (per 1,000 person-year) |  | 23.4 | 24.9 |  |  | 10.8 | 13.1 |  |
| Unadjusted HR (95% CI) |  | 1.00 (ref) | 1.05 (0.61–1.82) | 0.84 |  | 1.00 (ref) | 1.22 (0.75–1.99) | 0.42 |
| Adjusted HR (95% CI) |  | 1.00 (ref) | 1.24 (0.67–2.29) | 0.50 |  | 1.00 (ref) | 1.20 (0.71–2.03) | 0.48 |
|  |  |  |  |  |  |  |  |  |
| **Major cardiovascular events**^‡^ |  |  |  |  |  |  |  |  |
| No. of patients |  | 37 | 174 |  |  | 53 | 237 |  |
| Event rate (per 1,000 person-year) |  | 55.7 | 65.7 |  |  | 29.1 | 39.0 |  |
| Unadjusted HR (95% CI) |  | 1.00 (ref) | 1.18 (0.82–1.68) | 0.37 |  | 1.00 (ref) | 1.34 (0.99–1.80) | 0.05 |
| Adjusted HR (95% CI) |  | 1.00 (ref) | 1.26 (0.86–1.83) | 0.23 |  | 1.00 (ref) | 1.39 (1.01–1.90) | 0.04 |
|  |  |  |  |  |  |  |  |  |
| **Hospitalization for heart failure** |  |  |  |  |  |  |  |  |
| No. of patients |  | 38 | 204 |  |  | 31 | 171 |  |
| Event rate (per 1,000 person-year) |  | 61.6 | 84.5 |  |  | 17.2 | 28.9 |  |
| Unadjusted HR (95% CI) |  | 1.00 (ref) | 1.39 (0.98–1.96) | 0.06 |  | 1.00 (ref) | 1.67 (1.14–2.45) | 0.008 |
| Adjusted HR (95% CI) |  | 1.00 (ref) | 1.17 (0.81–1.70) | 0.39 |  | 1.00 (ref) | 1.74 (1.17–2.61) | 0.007 |

*Data are presented as number or hazard ratio (95% CI).

HFpEF, heart failure with preserved left ventricular ejection fraction; MI, myocardial infarction; β, beta blockers; CI, confidence interval; HR, hazard ratio.

^†^The primary outcome was a composite of cardiovascular death, aborted cardiac arrest, nonfatal myocardial infarction, nonfatal stroke, or hospitalization for the management of heart failure.

^‡^Major cardiovascular events included all-cause death, nonfatal myocardial infarction, and nonfatal stroke.

HFpEF, heart failure with preserved left ventricular ejection fraction; MI, myocardial infarction; β, beta blockers; CI, confidence interval; HR, hazard ratio.

**Supplemental Table 2. Baseline characteristics of propensity score-matched patients without myocardial infarction on and not on beta-blockers***

|  | β (−) | β (+) | Standardized | P value |  |
| --- | --- | --- | --- | --- | --- |
|  | n = 570 | n = 570 | Difference |  |  |
| Age (years) | 69.5 (9.9) | 69.5 (9.2) | 0.006 | 0.91 |  |
| Female sex (%) | 58.4 | 56.0 | 0.04 | 0.40 |  |
| Race and ethnicity (%) |  |  |  |  |  |
| White | 88.8 | 88.4 | 0.01 | 0.85 |  |
| Black | 8.4 | 9.0 | 0.01 | 0.75 |  |
| Asian | 1.1 | 0.9 | 0.01 | 0.76 |  |
| Others | 1.7 | 1.7 | <0.001 | 1.00 |  |
| Smoking status (%) |  |  |  |  |  |
| Never | 57.2 | 58.4 | 0.02 | 0.67 |  |
| Former | 33.5 | 32.5 | 0.02 | 0.70 |  |
| Current | 9.3 | 9.1 | 0.006 | 0.91 |  |
| Alcohol drinks/week (%) |  |  |  |  |  |
| 0 | 78.3 | 79.3 | 0.02 | 0.66 |  |
| 1–5 | 16.8 | 16.1 | 0.01 | 0.75 |  |
| 6–10 | 4.0 | 4.2 | 0.01 | 0.78 |  |
| 11– | 0.9 | 0.4 | 0.06 | 0.25 |  |
| NYHA functional classification (%) |  |  |  |  |  |
| I / II | 69.8 | 69.8 | <0.001 | 1.00 |  |
| III / IV | 30.2 | 30.2 | <0.001 | 1.00 |  |
| Body mass index (kg/m^2^)^†^ | 32.1 (7.7) | 32.5 (7.4) | 0.05 | 0.35 |  |
| Diabetes (%) | 29.5 | 32.1 | 0.05 | 0.33 |  |
| Hypertension (%) | 88.8 | 88.3 | 0.01 | 0.78 |  |
| Dyslipidemia (%) | 49.3 | 47.9 | 0.02 | 0.63 |  |
| History of cardiovascular events (%) |  |  |  |  |  |
| Angina pectoris | 34.2 | 35.8 | 0.03 | 0.57 |  |
| Stroke | 6.8 | 8.6 | 0.06 | 0.26 |  |
| Peripheral arterial disease | 6.7 | 6.7 | <0.001 | 1.00 |  |
| Hospitalization for heart failure | 70.5 | 71.9 | 0.03 | 0.60 |  |
| Atrial fibrillation | 37.5 | 36.3 | 0.02 | 0.66 |  |
| Percutaneous coronary intervention | 6.0 | 6.8 | 0.03 | 0.54 |  |
| CABG surgery | 4.7 | 4.4 | 0.01 | 0.77 |  |
| Implanted cardioverter defibrillator | 0.5 | 1.0 | 0.06 | 0.31 |  |
| Pacemaker | 8.1 | 7.9 | 0.006 | 0.91 |  |
| COPD (%) | 13.0 | 14.7 | 0.05 | 0.39 |  |
| Asthma (%) | 8.5 | 9.9 | 0.04 | 0.41 |  |
| Medications (%) |  |  |  |  |  |
| ACE-I / ARB | 84.9 | 86.1 | 0.03 | 0.55 |  |
| Calcium channel blockers | 50.0 | 49.3 | 0.01 | 0.81 |  |
| Diuretics | 82.5 | 80.9 | 0.04 | 0.49 |  |
| Aspirin | 54.9 | 52.6 | 0.04 | 0.44 |  |
| Statin | 35.3 | 32.8 | 0.05 | 0.38 |  |
| Randomization arm |  |  |  |  |  |
| Spironolactone (%) | 49.8 | 46.7 | 0.06 | 0.28 |  |
| Estimated GFR(mL/min/1.73 m^2^) | 68.7 (20.4) | 68.9 (21.8) | 0.005 | 0.92 |  |
| Systolic blood pressure (mmHg) | 129.8 (13.4) | 130.4 (13.8) | 0.04 | 0.44 |  |
| Diastolic blood pressure (mmHg) | 76.5 (10.4) | 77.0 (10.7) | 0.04 | 0.47 |  |
| Heart rate (beats per minute) | 69.8 (10.5) | 69.9 (10.5) | 0.01 | 0.81 |  |
| Region of enrollment (%) |  |  |  |  |  |
| United States | 29.8 | 29.6 | 0.004 | 0.94 |  |
| Canada | 9.1 | 7.9 | 0.04 | 0.45 |  |
| Russia | 27.4 | 28.8 | 0.03 | 0.59 |  |
| Republic of Georgia | 20.2 | 18.8 | 0.03 | 0.55 |  |
| Brazil | 7.9 | 9.3 | 0.05 | 0.39 |  |
| Argentina | 5.6 | 5.6 | <0.001 | 1.00 |  |
| Health state‡ | 60.4 (18.3) | 59.9 (18.2) | 0.02 | 0.65 |  |

*Data are presented as number of participants, percent, or mean (standard deviation).

^†^Body mass index was calculated as weight in kilograms divided by the square of height in meters.

‡Health state was assessed using a visual analog scale (0–100: the worst status corresponds to 0 and the best status corresponds to 100).

NYHA, New York heart association; CABG, coronary artery bypass graft; COPD, chronic obstructive pulmonary disease; ACE-I, angiotensin-converting enzyme inhibitors; ARB, angiotensin II receptor blockers; GFR, glomerular filtration rate; MI, myocardial infarction; β, beta blockers.
